# Genetic Characterization of *Streptococcus iniae* in Diseased Farmed Rainbow Trout (*Onchorhynchus mykiss*) in Iran

**DOI:** 10.1100/2012/594073

**Published:** 2012-04-01

**Authors:** A. Erfanmanesh, M. Soltani, E. Pirali, S. Mohammadian, A. Taherimirghaed

**Affiliations:** ^1^Academic Center of Education, Culture and Research (ACECR), Tehran, Iran; ^2^Department of Aquatic Animal Health, Faculty of Veterinary Medicine, University of Tehran, P.O. Box 14155-6453, Tehran, Iran

## Abstract

Genetic characterization of strains of *Streptococcus iniae* recovered from morbidity and mortality of farmed rainbow trout in different provinces of Iran were studied. The Gram-positive cocci isolates were obtained from the kidney tissues of diseased rainbow trout on blood agar at 25°C for 72 h. The grown bacteria were then characterized using biochemical and molecular works. The identified 26 isolates of *S. iniae* producing a 513 bp in PCR procedure were then compared using random amplified polymorphic DNA (RAPD) analysis using 9 random primers. The phylogenetic tree of the RAPD product using UPMGA software included these strains in one genetic group but into two clusters. The results of this study show that *S. iniae* strains from the diseased rainbow trout in the north part of Iran are genetically similar to those strains in the south and west parts of the country.

## 1. Introduction

*Streptococcus iniae* is not only one of the major causative agents of streptococcosis in aquaculture industry but also is an important zoonotic bacterial disease causing morbidity and mortality in humans [1–5]. The emergence of disease has occurred in a range of aquatic animals including many species of marine and freshwater of both wild and cultured environments [1, 2, 4, 6]. To date, the disease has been identified in almost all continents causing significant losses in several commercial fish species [1, 2]. The estimated annual impact of disease outbreaks by *S. iniae* in aquaculture sector of some countries was reported to be 100 million USD [1, 7]. In Iran, since its first report in rainbow trout farming, streptococcosis has caused significant losses in the aquaculture industry. A total annual loss due to this disease in trout farming has been estimated about 15 million USD [8]. Although adequate studies have focused on the immune-pathogenesis of the infection, minimal data is available on the genetic characterization particularly on genetic diversity of the isolated strains of this bacterium in fish [3, 9]. The importance of this is to provide an effective method of mass vaccination covering a number of isotypes and vaccination is one of the most feasible ways to prevent the losses due to this zoonotic bacterial disease in aquaculture industry [10]. Previoous work showed that it was possible to isolate the bacterium from different parts of Iran and recent attempts resulted in producing a local commercial vaccine inside the country [6, 8]. However, because of existing of heterogeneous strains of *S. iniae* [9], it is important to know the possible genetic diversity of the virulent isolates. Such data will assist to improve the efficacy and potency of the produced vaccines. Therefore, the aim of this study was to compare the recovered isolates of *S. iniae* at molecular level to determine if intraspecific variants could be found among the isolates from different geographical locations of Iran which is a big land with different climates and environmental conditions. 

## 2. Materials and Methods

### 2.1. Bacterial Isolates

A total of 60 isolates of Gram-positive cocci from the affected farmed trout at different geographical regions were used (Table 1). These isolates were recovered from the kidney tissues of diseased trout in states of Tehran, Lorstan, Charmahal-va-Bakhteyari, Gilan, Fars, and Mazandaran. Each bacterial isolate was recovered from at least five diseased trout showing clinical signs including bilateral exophthalmia, darkening of body, loss of appetite, and abdominal distention.

### 2.2. Bacteriological Methods

The bacterial isolates were recovered from the clinically diseased fish of different mass on tryptic soy agar supplemented with 5% defibrinated sheep blood at 25°C for 72 h. Presumptive identification of the isolates was made using traditional phenotypic methods.

### 2.3. Molecular Studies

Biospin Bacteria Genomic DNA Extraction Kit (Bioflux, Japan) was used for extraction of bacterial DNAs. The primers used in this study were F-GTCGTAACAAGGTAAGCCGTATCG and R-CTTACCTTAGCCCCAGTCTAACGAC [5] that identifiy 16–23S rRNA of the bacterial DNA giving a PCR product of 513 bp on a gel. PCR was performed with a Bio-Rad thermocyler (USA). A reaction matrix of 25 *μ*L contained 2.5 *μ*L PCR 10x buffer (Ferments, Lithuania), 30 pmol of each primer, 1.5 U Taq polymerase (DreamTaq, Ferments, Lithuania), 5 *μ*L dNTPs (Sinagen company, Iran, 0.2 mM) and 100 ng template DNA. Typical cycling parameters were: 1 min primary denaturation at 94°C, 1 min denaturation at 94°C, 1 min annealing at 45°C, and 1.5 min extension at 72°C for 35 cycles. The reaction was started by a denaturation step for 3 min at 94°C and ended with a 10 min extension step at 72°C. The PCR products were then electrophoreses using 2% gel agarose stained using Syber green (Sinagen company, Iran). *S. iniae* (a local strain collection with accession number: AF048773) was included as positive control and *Lactococcus garvieae* (a local strain collection with accession number X54262) as negative control.

### 2.4. Random Amplified Polymorphism DNA (RAPD)

For RAPD, 9 random primers were used (Table 2). A reaction mixture of 25 *μ*L contained 2.5 *μ*L 10x buffer (Fermentas), 1.5 *μ*L Taq polymerase, 30 pmol of each primer, 5 *μ*L dNTPs (0.2 mM), and 100 ng template DNA. The amplification cycles were 1 min denaturation at 94°C, 1 min annealing at 35°C, and 1.5 min extension at 72°C for 35 cycles. The reaction started by denaturation step for 3 min at 94°C and ended with a final extension for 10 min at 72°C. The amplified DNA fragments were electrophoresed in 1.5% agarose gel and stained by Syber green. The gels were photographed and the RAPD patterns of the bacterial isolates were compared. The phylogenic relationship of the isolated bacteria was drawn using the unweighted-pair group method with average linkage (UPMGA) by mega 4 software. 

## 3. Results

**Figure 1:**
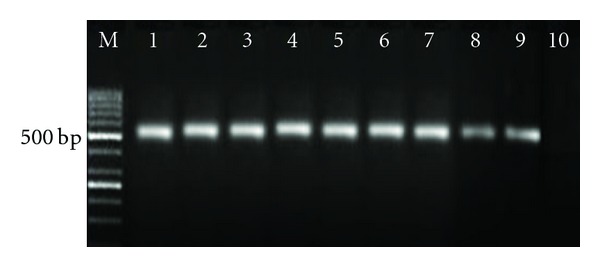
PCR product of *S. iniae* isolates obtained from diseased trout showing molecular weight of 513 bp on 2% agarose gel stained by Syber geen. M = marker, Lanes 1–8 = *S. iniae* isolates obtained from the diseased trout in Iran, Lane 9 = Positive control (*S. iniae*), Lane 10 = negative control (*L. gaarvieae*).

**Figure 2:**
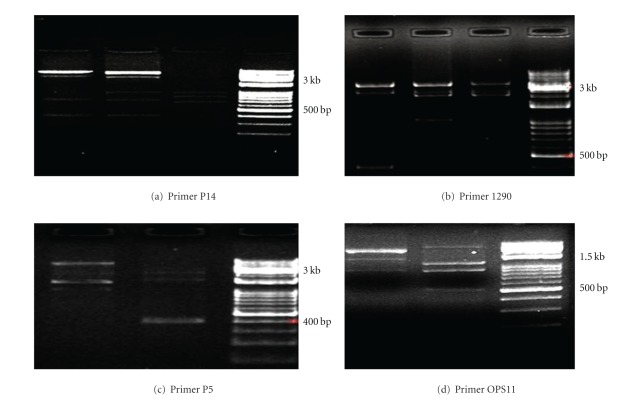
PCR gel RAPD products using primer p14 showing 3–5 bands with molecular weight 350–3000 bp (a), OPS11 primer showing 3-4 bands with molecular weight of 540–3290 bp (d), primer 1290 showing 3-4 bands with molecular weight of 400–3400 bp (b), primer p5 showing 3 bands with molecular weight of 400–4000 bp (c).

The biochemical profiles of the isolates included them into two groups *S. iniae* (26 isolates) and *L. garvieae* (34 isolates) (Table 3).* L. garvieae* utilized citrate, nitrate, lactose, and gelatin, while *S*. *iniae *isolates were positive for ornithine and mannitol. These 26 isolates were then subjected to PCR for further confirmation and the obtained results showed that all isolates were *S. iniae* giving a band of 513 bp for PCR products (Figure 1). Therefore, these *S. iniae* isolates were used for RAPD analysis. The banding patterns of each random primer are shown in Table 4. At most, five different RAPD banding patterns were observed (Table 4). The largest number of bands (five bands and three patterns) were observed using the primers P14 and1290 (four bands and three patterns) (Figures 2(a) and 2(b)), and the least banding patterns (three bands and one pattern) were seen using primer P4. Also, primers OPS11 and P5 resulted in production of 3-4 bands and two banding patterns (Figures 2(c) and 2(d)). Primers P1, P2, and P3 were able to produce only one band (Table 4), and thus, were not used for banding pattern analysis. The banding patterns were reproducible. The PCR was performed on all *S. iniae* isolates at two times and no difference was seen in the DNA pattern from one RAPD analysis to the next. The positive *S. iniae* strain was always included as an internal control for every PCR test to ensure that RAPD always produced the same DNA pattern as before.

**Figure 3:**
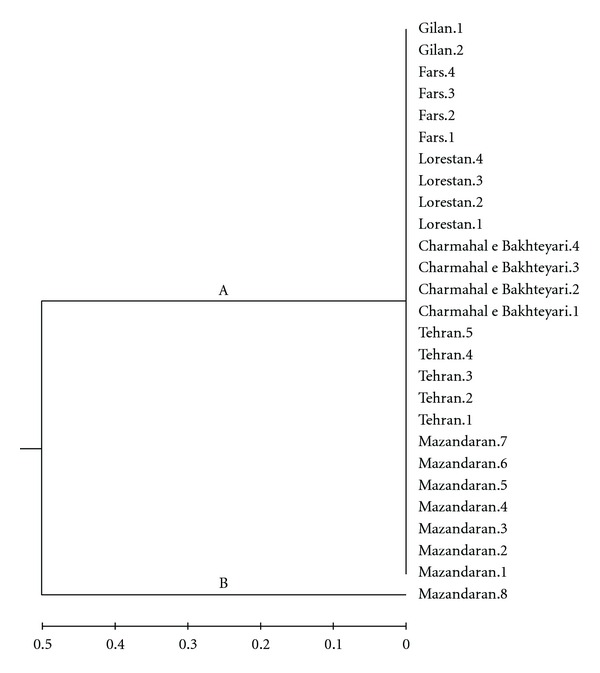
Phylogenetic tree of RAPD analysis of 26 isolates of *S. iniae* recovered from diseased trout in 6 states of Iran using UPGMA program showing one group and two clusters.

 When these data were subjected to UPMGA program, they were clustered into one group and two clusters (Figure 3). Only one strain of the bacterium from Mazandaran region was included in a separated cluster, while other isolates were included in one cluster.

## 4. Discussion 

Characterization of bacteria into known groups according to phenotypic features and virulence is an important tool to understanding the way for the identification and typing of pathogenic isolates. The morbidity and mortality due to *S. iniae* in aquaculture sector is now a big obstacle for having a sustainable aquaculture industry worldwide [1, 2]. As this bacterial agent is also a zoonotic microganism, this obstacle increases dramatically. The use of biochemical features for differentiation of the virulent strains of gram positive cocci including *S. inaie, S. parauberis, S. agalactiae, S. disagalactiae, *and* L. garvieae *is difficult because of the variable results and long time required [1, 2]. Therefore, using molecular works for the epidemiological purposes are essential to improve the preventive measures against the disease outbreaks in the fish farms. This is particularly important in case of disease prevention by vaccination methods. 

 Only a few works focused on the genetic features of *S. inaie* isolates in fish Eldar et al. (1997) [11] were able to show some genetic differences between some isolates of this bacterium obtained from diseased fish in the United state of America and Middle East using restriction length polymorphism of 16S rDNA of the bacterium. Although Dodson et al. (1999) [3] believed that it is possible to biochemically separate the pathogenic isolates from nonpathogenic isolates of *S. iniae* in fish, they could not distinguishe between either the invasive and noninvasive isolates in fish as well as between the fish and human isolates of *S. iniae* using the six different primers and RAPD analysis plus repeated PCR techniques. The *S. iniae* isolates used in their works were originally recovered from human (7 isolates), dolphin (1 isolate), and fish (38 isolates) in the United State of America (USA), Canada, and Middle East. However, Fuller et al. (2001) [12] reported that *S. iniae* virulence is associated with distinct genetic profile and demonstrated differences between pathogenic and nonpathogenic isolates. Also, Kvitt and Colorni (2004) [9] were able to separate 35 isolates of this bacterium recovered from the USA and Middle East into two groups using RAPD analysis. They found out that the trout isolates can be separated into one cluster which is different from other isolates recovered from other fish species including Asian sea bass (*Lates calcarifer*) and European sea bass (*Dicentrarchus labrax*). In the present study, all bacterial strains of *S. iniae* showed identical phenotypic features. However, using RAPD analysis, we could find up to 5 profiles/bands for the isolates recovered from diseased trout in 6 states of Iran. The phylogenetic analysis also included all bacterial strains into one group but into two seperated clusters. Therefore, it seems that virulence strains of *S. iniae* possess a high genetic similarity in trout aquaculture in Iran. If so, then for formulation of a whole inactivated vaccine, it is possible to induce an identical protection in fish using different isolates of this bacterium obtained from different regions of the country. However, more *in vivo* works are required to evaluate the efficacy of the produced vaccines by isolated bacteria from different parts of the country. Also, more works such as RNA sequencing are required to further genotypic characterization of these bacterial isolates of *S. iniae* in farmed rainbow trout in Iran. 

In conclusion, RAPD analysis of the Iranian isolates of *S. iniae* obtained from diseased trout in north west and south west of Iran gave high genetic similarity indicating a probable identical protective level against the disease in the studied states of the country. However, more studies are required to isolate and genetically characterize *S. iniae* strains from other parts of the country because of poor quarantine practice which allow the possible entrance of new and probably genetically different isolates of the bacterium through the frequently importation of large quantity of eyed-eggs of trout as well as several species of ornamental fish into the country.

## Figures and Tables

**Table 1:** Regional locations of the affected trout farms used for isolation of *S. inaie*.

State	Region/location	Number of bacterial isolates
Tehran	Jajrod, Damavand, Firozkoh	11
Gilan	Langroud	4
Mazandaran	Haraz, Marzanabad, Tonekabon	16
Lorstan	Alashtar, Aligodarz, Dorud	10
Charmahal-va-Bakhteyari	Sendegan, Bazoft, Samsami	11
Fars	Bayza, Dorudzan, Cheshmebonab	8

**Table 2:** Random primers used for RAPD analysis using 35°C as the annealing temperature.

Primer sequence	Location
GGTGCGGGAA	P1
GTTTCGCTCC	P2
GTAGACCCGT	P3
AAGAGCCCGT	P4
AACGCGCAAC	P5
CCCGTCAGCA	P6
GATCAAGTCC	P14
AGTCGGGTGG	OPS11
GTGGATGCGA	1290

**Table 3:** Phenotypic features of *Lactococcus garvieae *and *Streptococcus iniae *recovered from diseased rainbow trout in 6 states of Iran. Characters showing in parentheses are the published data [2, 5]. V = variable results; + = positive; − = negative; ? = Not defined.

Feature	*Lactococcus Garvieae*	* Streptococcus iniae*
Gram stain	+ (+)	+ (+)
Morphology	Cocci (cocci)	Cocci (cocci)
Hemolysis	*α*/*β* (*α*/*β*)	*α*/*β* (*α*/*β*)
Catalase	− (−)	− (−)
Oxidase	− (−)	− (−)
Motility	− (−)	− (−)
O/F	+/+ (+/+)	− (F)
Citrate	+ (−)	− (?)
NO3	+/− (−)	− (?)
Indole	− (?)	− (−)
MR/VP	+/− (+/−)	+/− (+/−)
Glucose	+ (+)	+ (+)
Lactose	+/− (+)	− (−)
Maltose	+ (+)	V (?)
Mannitol	(V)	+ (+)
Sucrose	V (?)	+ (+)
Inositol	− (−)	− (−)
Arabinose	− (−)	− (−)
Xylose	− (−)	− (−)
Lysine	− (−)	− (−)
Ornithine	− (−)	+ (?)
Arginine	+ (+)	+ (+)
Esculin	+ (+)	+ (+)
Urea	− **(?)**	− (?)
Gelatin	**+ (?) **	− (−)
H_2_S	−(−)	−(−)

**Table 4:** Banding patterns observed in *S. iniae* isolates using RAPD analysis. The numbers of different DNA banding profiles are listed with each primer used in the typing procedure. The P14 primer was able to discriminate between the isolates with the largest number of different banding patterns. — = indicating of no RAPD pattern.

Primer	Sequence (5′→3′)	Band size (bp)	Number of RAPD bands	Number of RAPD patterns
P14	GATCAAGTCC	3000–350	3–5	3
Ops11	AGTCGGGTGG	3290–540	3-4	2
P5	AACGCGCAAC	4000–400	3	2
1290	GTGGATGCGA	3400–400	3-4	3
P4	AAGAGCCCGT	3000–1550	3	1
P1	GGTGCGGGAA	3290–2500	2	—
P2	GTTTCGCTCC	1400	1	—
P3	GTAGACCCGT	3000	1	—
P6	CCCGTCAGCA	1200	1	—
